# SMAP2 Regulates Retrograde Transport from Recycling Endosomes to the Golgi

**DOI:** 10.1371/journal.pone.0069145

**Published:** 2013-07-08

**Authors:** Tatsuyuki Matsudaira, Yasunori Uchida, Kenji Tanabe, Shunsuke Kon, Toshio Watanabe, Tomohiko Taguchi, Hiroyuki Arai

**Affiliations:** 1 Department of Health Chemistry, Graduate School of Pharmaceutical Sciences, University of Tokyo, Tokyo, Japan; 2 Medical Research Institute, Tokyo Women’s Medical University, Tokyo, Japan; 3 Department of Molecular Immunology, Institute of Development, Aging and Cancer, Tohoku University, Sendai-shi, Miyagi, Japan; 4 Department of Biological Science, Graduate School of Humanities and Sciences, Nara Women’s University, Nara-shi, Nara, Japan; 5 Pathological Cell Biology Laboratory, Graduate School of Pharmaceutical Sciences, University of Tokyo, Tokyo, Japan; Institut Curie, France

## Abstract

Retrograde transport is where proteins and lipids are transported back from the plasma membrane (PM) and endosomes to the Golgi, and crucial for a diverse range of cellular functions. Recycling endosomes (REs) serve as a sorting station for the retrograde transport and we recently identified evection-2, an RE protein with a pleckstrin homology (PH) domain, as an essential factor of this pathway. How evection-2 regulates retrograde transport from REs to the Golgi is not well understood. Here, we report that evection-2 binds to SMAP2, an Arf GTPase-activating protein. Endogenous SMAP2 localized mostly in REs and to a lesser extent, the trans-Golgi network (TGN). SMAP2 binds evection-2, and the RE localization of SMAP2 was abolished in cells depleted of evection-2. Knockdown of SMAP2, like that of evection-2, impaired the retrograde transport of cholera toxin B subunit (CTxB) from REs. These findings suggest that evection-2 recruits SMAP2 to REs, thereby regulating the retrograde transport of CTxB from REs to the Golgi.

## Introduction

Newly synthesized proteins that are destined for secretion or for residence within organelles move from the endoplasmic reticulum (ER), through the Golgi, then to their final destination [1]. . This membrane outflow is counteracted by retrograde membrane flow that originates from either PM or endosomal system [2,3]. Golgi proteins, such as TGN38/46, GP73, mannose 6-phosphate receptors, and furin, utilize retrograde membrane transport to maintain their predominant Golgi localization [4–9]. Intriguingly, some protein toxins produced by bacteria and plants, e.g., cholera toxin, Shiga toxin, and ricin, exploit this retrograde transport to reach the Golgi/ER then the cytosol, where they exert their toxicity [10–12].

REs serve as an important sorting station in the retrograde pathway. CTxB and Shiga toxins pass through REs before they reach the Golgi [13–15]. We recently found that evection-2, an RE protein that contains an N-terminal PH domain and a C-terminal hydrophobic region, plays an essential role in retrograde transport [13]. In cells depleted of evection-2, the retrograde transport of CTxB to the Golgi was impaired in REs, and the Golgi localization of TGN46 and GP73 was abolished. Evection-2 specifically binds phosphatidylserine (PS) through its PH domain [13,16], and this interaction is required for the function of evection-2 and its localization to REs where PS is highly enriched. The molecular mechanism of how evection-2 regulates retrograde transport is not well understood.

ADP-ribosylation-factors (Arfs) belong to the Ras superfamily of GTP-binding proteins switching between the GTP- and GDP-bound forms [17–19]. Arfs are involved in membrane trafficking, actin remodeling, and phospholipid metabolism. Arf-specific GTPase-activating proteins (Arf GAPs) regulate Arfs by stimulating their slow intrinsic GTP hydrolysis [18–20]. In humans, Arf GAPs are classified according to their domain structure into 10 subfamilies including 31 members and are characterized by the presence of a zinc finger motif. The SMAP subfamily consists of two members, SMAP1 and SMAP2 [21,22]. Human SMAPs are about 50 kD and lack other defined domains, thus the acronym s mall Arf GAP protein. SMAPs have been implicated as regulators of endocytosis. SMAP1 functions in clathrin-dependent endocytosis at the PM [21]. SMAP2, when exogenously expressed, co-localized with clathrin at perinuclear area (a TGN marker), partially co-localized with transferrin receptor (TfnR) (an early/recycling endosomal marker), and impaired the retrograde transport of a CD25-TGN38 chimera protein from PM to TGN [22].

In the present study, we report that endogenous SMAP2 localizes mostly in REs and is essential for the retrograde transport of CTxB from REs to the Golgi. SMAP2 binds evection-2 and the RE localization of SMAP2 is abolished in cells depleted of evection-2. These findings suggest that evection-2 recruits SMAP2 to REs, thereby regulating the retrograde transport of CTxB from REs to the Golgi.

## Materials and Methods

### Plasmids

Myc-tagged evection-2 and FLAG-tagged evection-2 constructs were previously described [13].

### Reagents

Mouse anti-EEA1, anti-GM130, anti-Lamp1, and anti-Rab11 antibodies were purchased from BD Biosciences. Mouse anti-α-tubulin antibody, anti-Myc antibody (9E10), and rabbit anti-SMAP2 antibody were purchased from SIGMA. Rabbit anti-FLAG antibody was purchased from Cell Signaling Technology. Mouse anti-TfnR antibody was purchased from Zymed Laboratories. Mouse anti-CD63 antibody was purchased from Cymbus Biotechnology. Rabbit anti-Syntaxin 5 antibody was purchased from Synaptic Systems. Sheep anti-TGN46 antibody was purchased from Serotec. Goat anti-VPS26 antibody was purchased from Everest Biotech. Rabbit anti-EGFR antibody, sheep anti-GP73 antibody, and donkey anti-goat IgG antibody-HRP were purchased from Santa Cruz Biotechnologies. Sheep anti-mouse IgG antibody-HRP and donkey anti-rabbit IgG antibody-HRP were purchased from GE Healthcare. Alexa-594 CTxB and Alexa-conjugated secondary antibodies were purchased from Invitrogen. Human holo-Tfn (Sigma) was conjugated with Alexa 488 using Alexa Flour succinimidyl ester (Invitrogen), and purified by PD-10 desalting columns (GE Healthcare).

### Cell culture

COS-1 cells (American Type Culture Collection) were cultured at 37°C with 5% CO_2_ in DMEM containing 10% heat-inactivated fetal calf serum, 100 units/ml penicillin, 100 µg/ml streptomycin, and 0.29 mg/ml glutamine.

### RNA interference

siRNA duplex oligomers used were as follows: GAUCCCAGACGCCUCAAAU (SMAP2 siRNA#1), GUUGUAUAUUAGGCAAACA (SMAP2 siRNA#2), CUGCAUGCUCCAGAUUGUU (evection-2 siRNA) from NipponEGT; HSS127678 (SMAP2 siRNA#3) from Invitrogen. Control siRNA (*Silencer* Negative Control no. 1 siRNA) was obtained from Ambion. A total of 20 nM siRNA was introduced to COS-1 cells using Lipofectamine RNAiMAX (Invitrogen) according to the manufacturer’s instruction.

### Immunocytochemistry

Cells were washed with PBS briefly, fixed with 4% PFA in PBS at room temperature or 10% trichloroacetic acid at 4°C for 15 min, and permeabilized with 0.1% Triton X-100 in PBS (for 5 min) or 0.1% saponin in PBS (for 10 min) for Lamp1 staining at room temperature. Blocking was performed with 3% BSA in PBS at room temperature for 30 min. Cells were then incubated with primary antibodies diluted in *Can Get Signal* immunostain Solution A (TOYOBO) at 4°C for 12-16 h. After washing three times with PBS, cells were incubated with secondary antibodies at room temperature for 1 h, washed with PBS, and mounted in PermaFluor (Thermo).

### CTxB uptake

Cells were washed with PBS briefly and pulsed with 1 µg/ml Alexa 594-CTxB for 3 min. After washing twice with PBS, cells were chased in the medium without CTxB for the indicated times.

### Tfn uptake and recycling assay

Cells were serum-starved for 30 min in DMEM and then incubated with 500 µg/ml Alexa488-Tfn at 37°C for 5 min. The cells were then washed twice with PBS and incubated for 15 or 60 min at 37°C prior to fixation.

### Cross-linking and immunoprecipitation

COS-1 cells grown on 100-mm dishes were transfected with FLAG-evection-2 or empty vector (pFLAG-CMV2) using Lipofectamine 2000 (Invitrogen). Twenty-four hours after transfection, cells were scraped in 1 ml of immunoprecipitation buffer composed of 25 mM Hepes-NaOH (pH 7.3), 150 mM NaCl, 1 mM EDTA, and protease inhibitor cocktail (Roche) and then homogenized using a ball-bearing cell homogenizer (4 passages, clearance 10 µm, Isobiotec, Germany). The cell homogenates were spun at 1,000 g for 5 min at 4°C to obatin post-nuclear supernatant (PNS). PNS was then lysed by adding Triton X-100 to a final concentration of 1%, followed by centrifugation at 12,000 g for 20 min at 4°C. The resultant supernatants were incubated for 2.5 h at 4°C with anti-FLAG M2 affinity gel beads (SIGMA). The beads were washed four times with immunoprecipitation buffer. Immunoprecipitated proteins were analysed by western blotting.

For crosslinking, PNS was incubated with dithio-bis(succinimidyl propionate) (DSP) for 2 h on ice. DSP was then quenched with 20 mM Tris-HCl (pH 7.4) for 15 min prior to solubilization.

### Confocal microscopy

Confocal microscopy was performed using a laser scanning microscope (model LSM 510 META, Carl Zeiss Microimaging) with a 63 x 1.4 Plan-Apochromat oil immersion lens. Excitation was done with a 30 mW Diode laser at 405 nm, a 30 mW argon laser emitting at 488 nm, and a 1.0 mW helium/neon laser emitting at 543 nm. Emissions were collected using a 420-480 nm band-pass filter for DAPI, a 505-530 nm band-pass filter for Alexa 488, and a META detector from 561 to 615 nm for Alexa 594.

### Western blotting

Proteins were separated by 12% polyacrylamide gel and then transferred to polyvinylidene difluoride membranes (Millipore). The membranes were blocked for 1 h using 10 mM Tris-HCl (pH 7.4)/150 mM NaCl/0.05% Tween-20/5% skim milk. The membranes were incubated with primary antibodies, followed by secondary antibodies conjugated to peroxidase. The proteins were visualized by chemiluminescence using ImageQuant LAS4000 (GE Healthcare).

### Statistical analysis

Statistical analysis was performed using Student’s two-tailed *t*-test.

### Fluorescent image analysis

Quantitation of images was performed with ImageJ software (NIH). Fluorescence intensity and Pearson coefficient were determined with RGB Profile Plot and JACoP plugin, respectively.

## Results

### Endogenous SMAP2 localized mostly in REs

**Figure 1:**
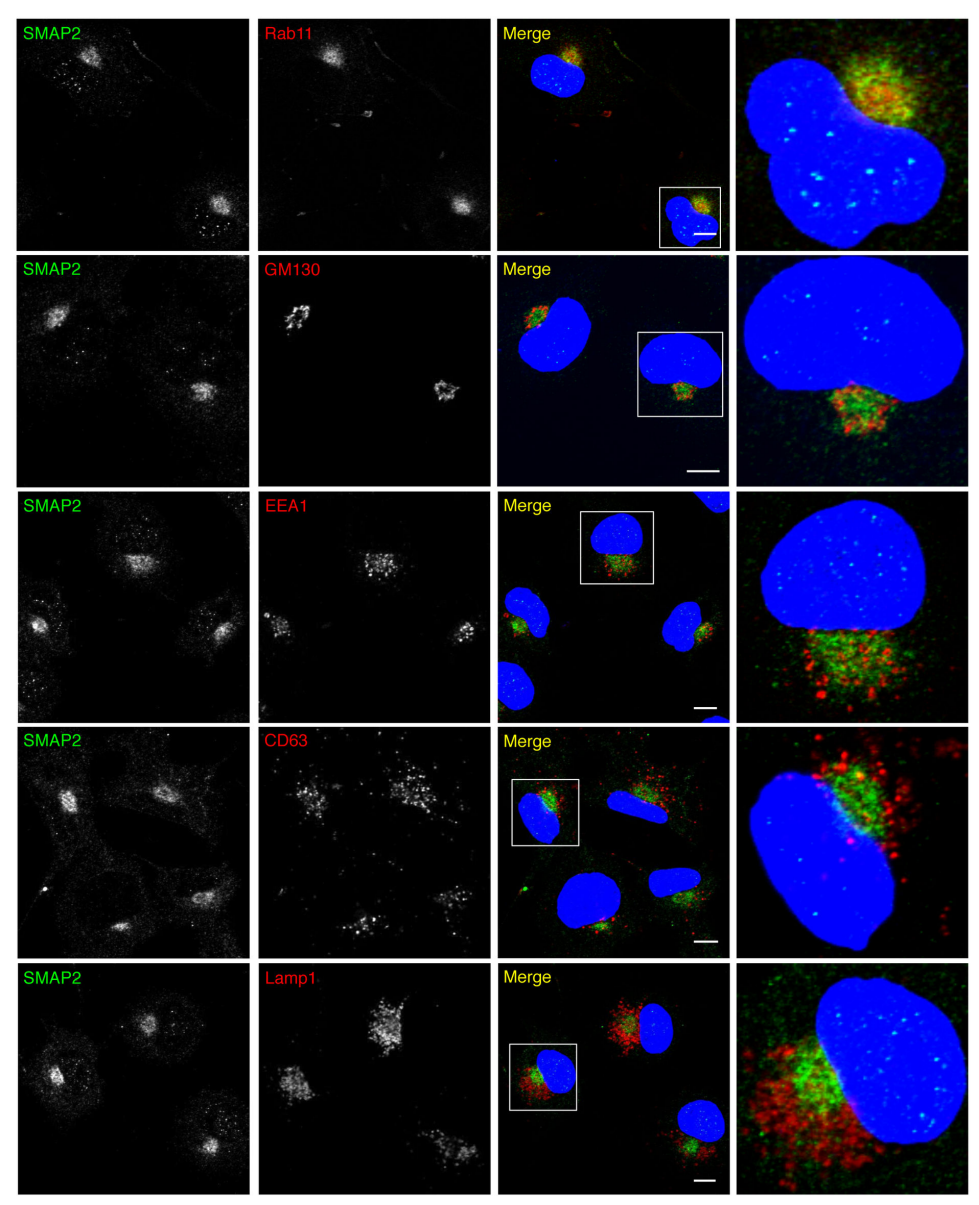
RE localization of endogenous SMAP2. COS-1 cells were fixed with TCA, permeabilized, and then stained for SMAP2 (green) and the indicated organelle markers (red). Nuclei were stained with DAPI (blue). Magnified images of boxed areas around the perinulcear region are shown in the right column. Scale bars, 10 µm.

The subcellular localization of endogenous SMAP2 was examined by immunolabeling in COS-1 cells, a cell line with distinctly separate organelles [23]. The Golgi, including the TGN, exhibits a ring-shape appearance, and REs are spatially confined within the Golgi. Early endosomes (EEs), late endosomes (LEs), and lysosomes are excluded from inside the Golgi (Figure S1). Among several combinations of fixation and permeabilization reagents tested, we found that fixation with trichloroacetic acid (TCA) and permeabilization with saponin or Triton X-100 yielded a decent signal of SMAP2. SMAP2 co-localized well with Rab11 (an RE protein), but not with GM130 (a Golgi protein), EEA1 (an EE protein), CD63 (a LE protein), and Lamp1 (a lysosomal protein), showing the preferential localization of endogenous SMAP2 in REs (Figure 1)

Specificity of the SMAP2 staining was confirmed by using a knockdown approach. Three unique siRNAs were designed based on human SMAP2 sequence and transfected into COS-1 cells. Seventy-two hours post-transfection, SMAP2 expression was effectively reduced (Figure S2A) and the perinuclear staining of SMAP2 was abolished (Figure S2B).

### Distribution of SMAP2 within REs

**Figure 2:**
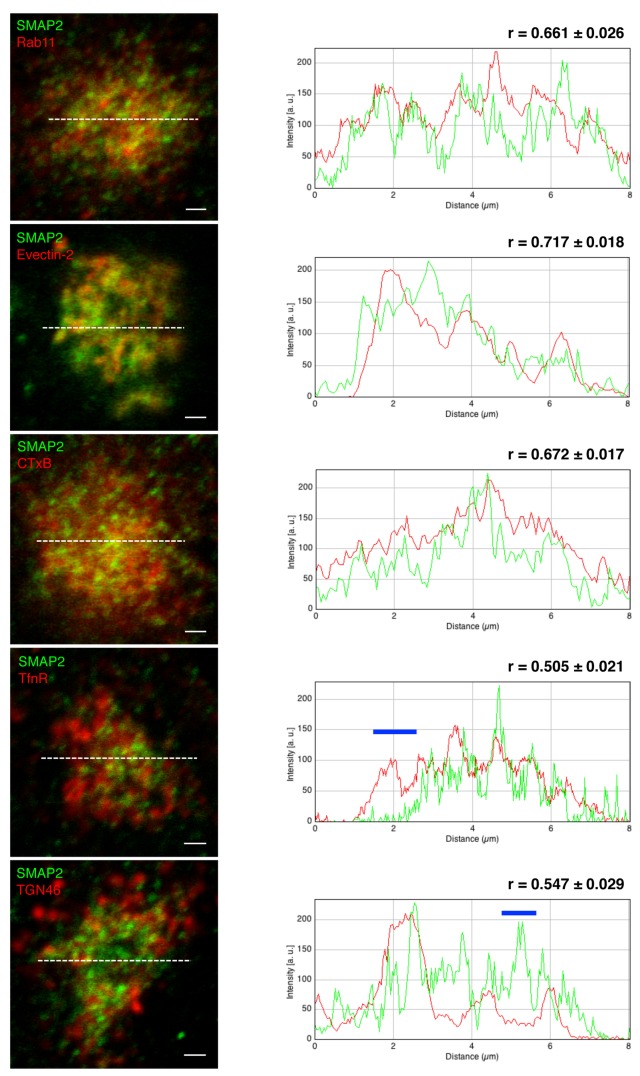
Co-localization analysis of SMAP2 with several RE/TGN proteins. COS-1 cells were fixed with TCA, permeabilized, and then stained for SMAP2 (green) and the indicated proteins (red). For evection-2, Myc-tagged evection-2 was transiently expressed and stained with anti-Myc antibody. For CTxB, cells were pulsed for 3 min with Alexa 594-CTxB, chased for 35 min at 37°C, and fixed. Fluorescence intensity profile along white dotted lines is shown in the right column. Blue lines in the graph indicate regions where SMAP2 do not co-localize with TfnR or TGN46. Pearson coefficient was obtained using multiple images (n > 12 cells). Data represent mean ± SD. Scale bars, 1 µm.

We next examined in detail the distribution of SMAP2 within REs. RE proteins (Rab11, TfnR, and evection-2) and a retrograde cargo (CTxB) that passes through REs were used as RE references. Since the TGN is in close proximity to the REs, a TGN protein (TGN46) was also examined. To quantitate the degree of co-localization of these proteins with SMAP2, Pearson coefficients were obtained with ImageJ using multiple images (n > 12). Among the RE references examined, Rab11, evection-2 showed relatively high Pearson coefficients (> 0.65) with SMAP2 (Figure 2). The Pearson coefficients (n > 10) between CTxB and SMAP2 varied at different chase periods (a 15-min chase, 0.563 ± 0.022; a 25-min chase, 0.620 ± 0.019; a 35-min chase, 0.672 ± 0.017), indicating that the co-localization of CTxB with SMAP2 increased gradually during CTxB stay in REs. Fluorescence intensity line scan profiles corroborated relatively similar distributions of these proteins with SMAP2. In contrast, TfnR and TGN46 showed smaller Pearson coefficients (< 0.55) with SMAP2. As indicated by blue bars in line scan profiles, there were regions where SMAP2 did not co-localize at all with TGN46 or TfnR. Together, these data showed the uneven distribution of SMAP2 within REs.

### SMAP2 knockdown impaired the retrograde transport of CTxB from REs

Two endosomal transport pathways are known to pass through REs [13,24]: one is a recycling pathway that transport cargoes, such as transferrin (Tfn)/TfnR, from REs to the PM and the other is a retrograde pathway that transport cargoes, such as CTxB, from REs to the Golgi. RE localization of endogenous SMAP2 motivated us to examine whether SMAP2 is involved in either of these pathways.

**Figure 3:**
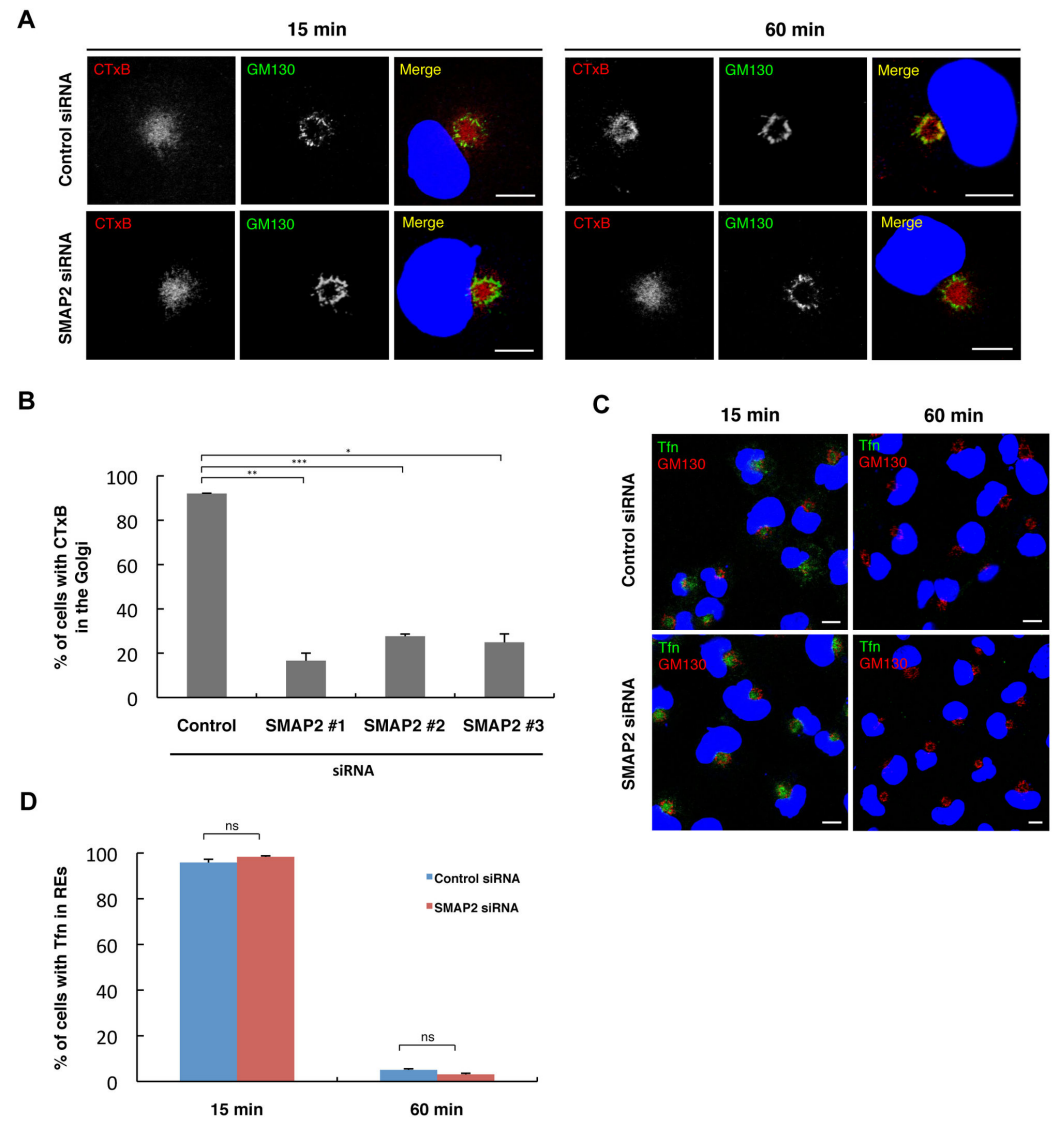
Knockdown of SMAP2 impaired the retrograde transport of CTxB from REs. (A) Cells treated with control siRNA (left column) or SMAP2 siRNA#1 (right column) were pulsed for 3 min at 37°C with Alexa594-CTxB and chased for the indicated times. Cells were then fixed, permeabilized, and stained for GM130. Nuclei were stained with DAPI (blue). (B) Cells in (A) were examined visually if CTxB reached the Golgi. The percentage of cells in which CTxB reached the Golgi after a 60-min chase was shown. Data represent mean ± SD, n > 150 cells. *P < 0.05, **P < 0.01, ***P < 0.001, Student’s two-tailed *t* test. (C) Cells treated with control or SMAP2 siRNA#1 were pulsed for 5 min at 37°C with Alexa488-Tfn and chased for the indicated times. Cells were then fixed, permeabilized, and stained for GM130. Nuclei were stained with DAPI (blue). (D) Cells in (C) were examined visually if Tfn was in REs. The percentage of cells in which Tfn was in REs at the indicated times was shown. Data represent mean ± SD, n > 70 cells. ns, not significant, Student’s two-tailed *t* test. Scale bars, 10 µm.

We first examined the effect of SMAP2 depletion on CTxB transport. In control cells, as shown previously [13], CTxB accumulated in REs after a 15-min chase, and showed a good co-localization with GM130 after a 60-min chase, confirming the CTxB transport from the PM through REs to the Golgi (Figure 3A). In cells depleted of SMAP2 with siRNA#1, the transport of CTxB from REs to the Golgi, not from the PM to REs, was significantly impaired. After a 60-min chase, CTxB still accumulated in REs, and did not co-localize with GM130 (Figure 3A). By counting the number of cells in which CTxB did not reach the Golgi, more than 80% of the cells depleted of SMAP2 with siRNA#1 had the defect of the CTxB transport from REs to the Golgi (Figure 3B). The other two siRNAs #2 and #3 also impaired the CTxB transport (Figure 3B).

**Figure 4:**
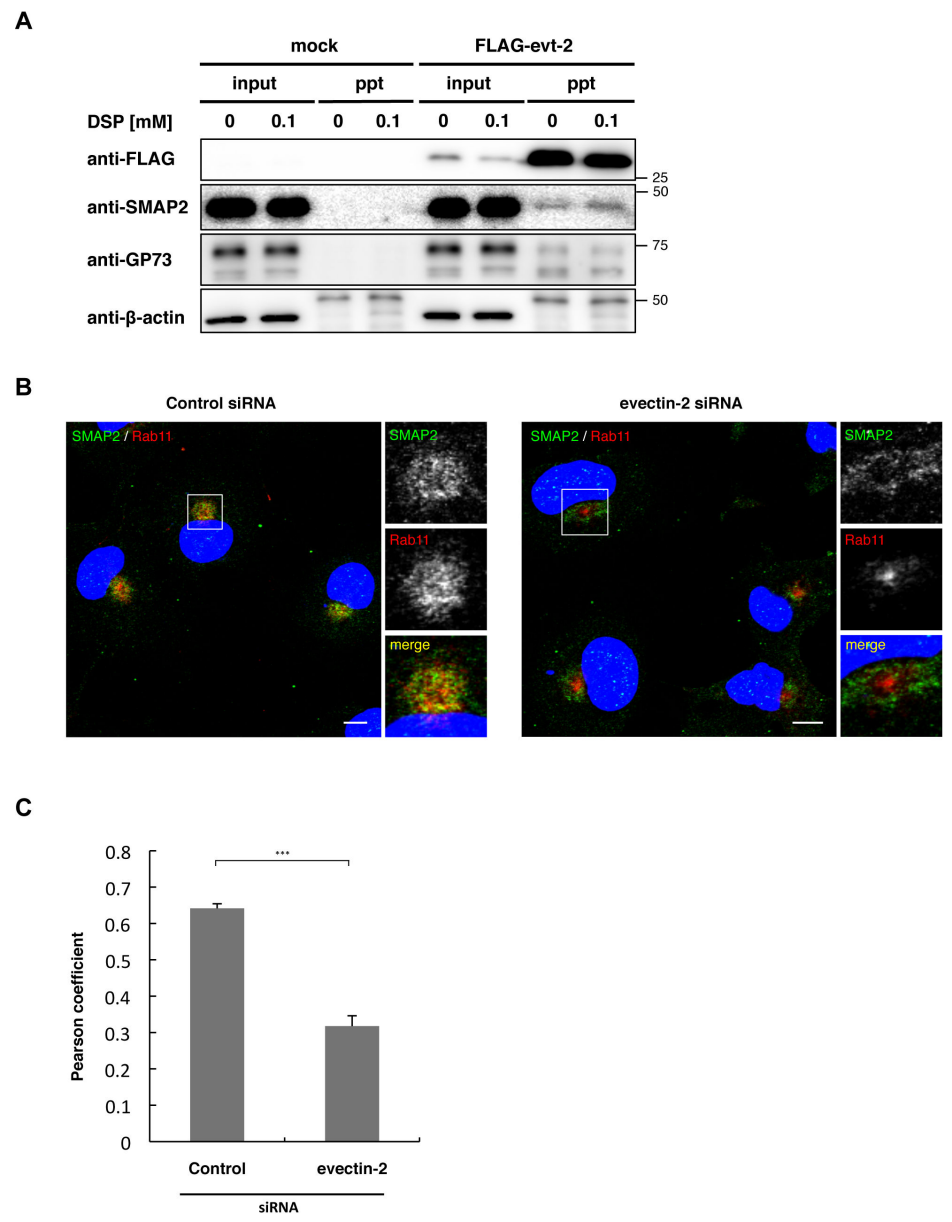
SMAP2 interacts with evection-2. (A) Flag-tagged evection-2 was expressed in COS-1 cells for 24 h. The cell homogenates were treated with 0 or 0.1 mM DSP and then solubilized by adding 1% Triton X-100. The lysates were then immunoprecipitated with anti-FLAG antibody. Immunoprecipitates were separated by SDS-PAGE, and then blotted with anti-FLAG, anti-SMAP2, anti-GP73, or anti-β-actin antibody. A 50-kDa band observed in the β-actin blot was IgG heavy chain. (B) Cells treated with control siRNA or evection-2 siRNA for 48 h were fixed, permeabilized, and stained for SMAP2 and Rab11. Nuclei were stained with DAPI (blue). Scale bars, 10 µm. (C) Pearson coefficient between SMAP2 and Rab11 in (B). Data represent mean ± SD, n > 16 cells. ***P < 0.001, Student’s two-tailed *t* test.

We next examined the effect of SMAP2 depletion on Tfn recycling pathway. In control cells, Alexa488-labeled Tfn accumulated in REs after a 15-min chase, and disappeared from the cells after a 60-min chase due to Tfn recycling back to the PM (Figure 3C and 3D). Cells depleted of SMAP2 showed the same Tfn kinetics as control cells (Figure 3C and 3D). Alexa488-labeled Tfn also accumulated in REs after a 15-min chase, and disappeared from the cells after a 60-min chase.

In summary, these results showed the selective involvement of SMAP2 in membrane transport through REs: SMAP2 is required for the retrograde transport of CTxB from REs to the Golgi, but not for the transport of Tfn from the PM to REs and from REs to the PM.

### Interaction of SMAP2 with evection-2

We previously showed that an RE protein, evection-2, is essential for the retrograde transport of CTxB from REs to the Golgi, but not required for Tfn recycling from REs to the PM [13]. The present findings that SMAP2 co-localized well with evection-2 (Figure 2) and that SMAP2 was essential for the retrograde transport of CTxB from REs to the Golgi, but not required for the Tfn recycling (Figure 3), led us to examine biochemical interaction of SMAP2 with evection-2.

FLAG-tagged evection-2 was expressed in COS-1 cells for 24 h. Cell lysates with detergent were then immunoprecipitated with anti-FLAG antibody. Immunoprecipitates were separated by SDS-PAGE and then blotted with anti-SMAP2 antibody. GP73 was used as a positive control, since evection-2 binds GP73 [13]. As shown in Figure 4A, SMAP2, along with GP73, were co-immunoprecipitated specifically from the lysates of cells that express FLAG-evection-2. Addition of a cross-linking reagent (DSP) to cell homogenates before detergent lysis increased the amount of SMAP2 that was co-immunoprecipiated with FLAG-evection-2. This result indicates that interaction of evection-2 with SMAP2 is susceptible to the detergent extraction.

We next examined the effect of evection-2 knockdown on SMAP2 localization. In cells treated with control siRNA, SMAP2 co-localized with an RE protein Rab11 (Pearson coeffieicnt 0.642±0.013) (Figure 4B and 4C). In contrast, in cells depleted of evection-2, SMAP2 and Rab11 segregated significantly. SMAP2 showed a doughnut-like distribution near the nucleus, with Rab11 confined to the center. The segregation of SMAP2 from Rab11 upon evection-2 knockdown was supported by the reduction of Pearson coefficient to 0.318±0.028. Therefore, evection-2 is essential for the RE localization of SMAP2. The organelle where SMAP2 relocalized to upon evection-2 depletion was not elucidated.

## Discussion

We previously identified evection-2 as an RE protein that is essential for retrograde transport from REs to the Golgi [13]. In the present study, we identified a second protein SMAP2, an Arf GAP, which functions in the retrograde transport of CTxB as evection-2. Furthermore, we found that evection-2 binds SMAP2 and is essential for the SMAP2 localization to REs.

Although SMAP2 localized in REs, its distribution over the REs was not even (Figure 2). SMAP2 showed a high degree of co-localization with evection-2 and CTxB (a retrograde cargo), but a lesser degree of co-localization with TfnR (a recycled cargo). As shown by the blue bar in a fluorescence intensity line scan profile (Figure 2: SMAP2 and TfnR), there was a region where TfnR did not co-localize at all with SMAP2. These observations suggested the two functionally separable subdomains in REs: one is a domain in which evection-2 and SMAP2 localize, functioning in the retrograde transport from REs to the Golgi and the other is a domain in which TfnR localizes, functioning the recycling transport from REs to the PM. In polarized cells, the PM contains distinct apical and basolateral domains [25,26]. The REs in polarized cells function to maintain the PM integrity by recycling membrane proteins back to the correct PM domains [27]. It is reported that the REs in polarized cells have subdomains where apical and basolateral cargoes are segregated [28]. Our study suggests that REs has also subdomains in non-polarized cells. Therefore, the existence of subdomains in REs may be a general feature of RE membranes, which facilitates the correct sorting of different cargo proteins.

Three Arf GAPs (ACAP1, ACAP2, and AGAP2), besides SMAP2, are known to localize in endosomes or function in endosomal membrane transport. ACAP1 localizes in REs and is essential for the recycling pathway from REs to the PM through the recognition of specific sequences in recycled cargoes, such as TfnR [29]. ACAP2 localizes to REs in PC12 cells stimulated with nerve growth factor, and regulates the neurite outgrowth [30]. AGAP2 is required for the exit of Shiga toxin B subunit (STxB) from EEs in HeLa cells [31]. Combined with the previous findings, the current study postulates that a network of endosomal pathways into or out of REs can be regulated by a network of endosomal Arf GAPs. A recent study in BSC-1 cell has shown that the retrograde transport of STxB proceeds from PM to EEs to REs then to the Golgi [15]. Thus, the retrograde transport of STxB from EEs to the Golgi may be dissected into two steps by two Arf GAPs. One is an AGAP2-dependent pathway, which corresponds to EEs to REs. The other is a SMAP2-dependent pathway, which corresponds to REs to the Golgi.

The Golgi proteins TGN46 and GP73 utilize retrograde membrane transport to maintain their predominant Golgi localization [5–7]. In cells depleted of SMAP2, both proteins still localized at the Golgi (Figure S3), indicating that SMAP2 does not contribute to the retrograde transport of TGN46 and GP73. Thus, SMAP2 showed specificity for retrograde cargoes: SMAP2 is selectively involved in the transprot of CTxB. Whether SMAP2 is involved in retrograde transport of cellular proteins remains to be determined. The apparent no-effect on TGN46 and GP73 localization upon SMAP2 depletion highly contrasted with the effect of evection-2 depletion, where TGN46 and GP73 redistributes from the Golgi to endosomal compartments [13]. Therefore, evection-2 requires SMAP2 for the retrograde transport of CTxB, but not that of TGN46 and GP73. Evection-2 might use a different Arf GAP to regulate the retrograde transport of TGN46 and GP73.

## Supporting Information

Figure S1 Schematic illustration of organelle distribution in COS-1 cells. The Golgi exhibits a ring-shape appearance, and REs are spatially confined within the Golgi. EEs, LEs, and lysosomes (gray objects) are excluded from inside the Golgi.
(PDF)

Figure S2 Validation of knockdown efficiency with SMAP2 siRNAs. (A) COS-1 cells were treated with control siRNA or SMAP2 siRNA#1, #2, #3 for 72 h. Cell lysates were prepared and then immunoblotted with anti–SMAP2 antibody. As a loading control, α-tubulin was used. (B) Cells treated with control siRNA or SMAP2 siRNA#1 were fixed, permeabilized, and stained for SMAP2.
(PDF)

Figure S3 Effect of SMAP2 knockdown on subcellular localization of several organelle markers. Cells treated with control siRNA or SMAP2 siRNA#1 for 72 h were fixed with PFA, permeabilized, and stained for GM130 (red), syntaxin 5 (red) or the indicated organelle markers (green). Scale bars, 10 µm.
(PDF)
