# Supplementary figures and images for: SMAP2 Regulates Retrograde Transport from Recycling Endosomes to the Golgi
Source: PLoS One. 2013 Jul 8;8(7):e69145. doi: 10.1371/journal.pone.0069145 (PMC3704519; doi:10.1371/journal.pone.0069145)

Figure S1

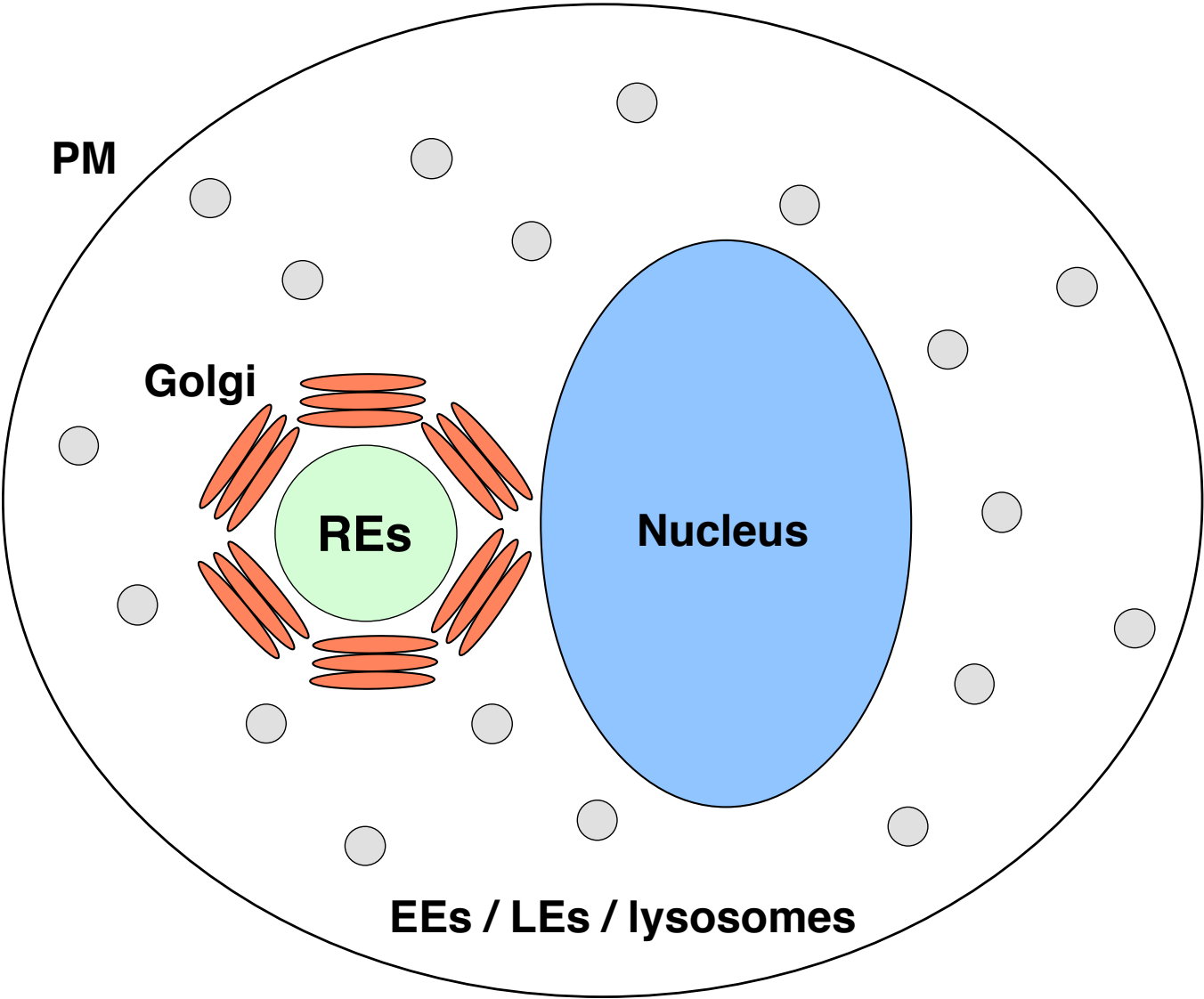

Supplement: Figure S1 — The Golgi exhibits a ring-shape appearance, and REs are spatially confined within the Golgi. EEs, LEs, and lysosomes (gray objects) are excluded from inside the Golgi. (PDF) [file pone.0069145.s001.pdf]

Figure S2

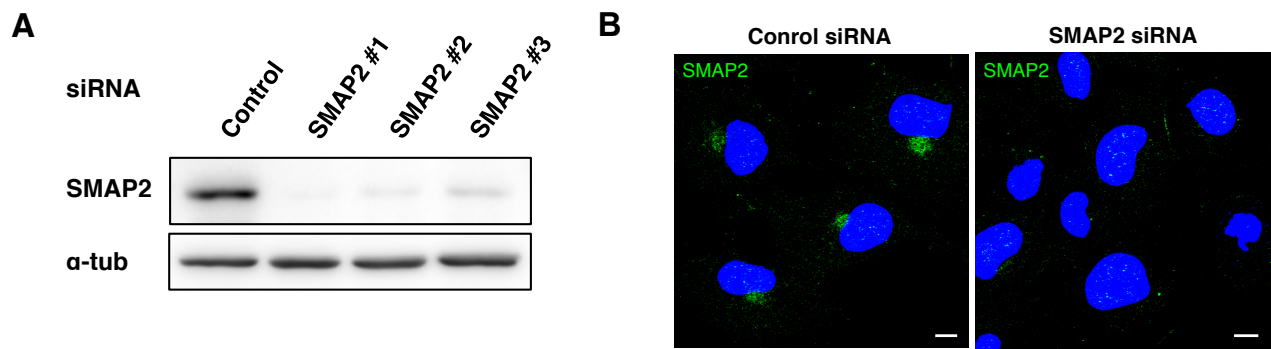

Supplement: Figure S2 — (A) COS-1 cells were treated with control siRNA or SMAP2 siRNA#1, #2, #3 for 72 h. Cell lysates were prepared and then immunoblotted with anti–SMAP2 antibody. As a loading control, α-tubulin was used. (B) Cells treated with control siRNA or SMAP2 siRNA#1 were fixed, permeabilized, and stained for SMAP2. (PDF) [file pone.0069145.s002.pdf]

Figure S3

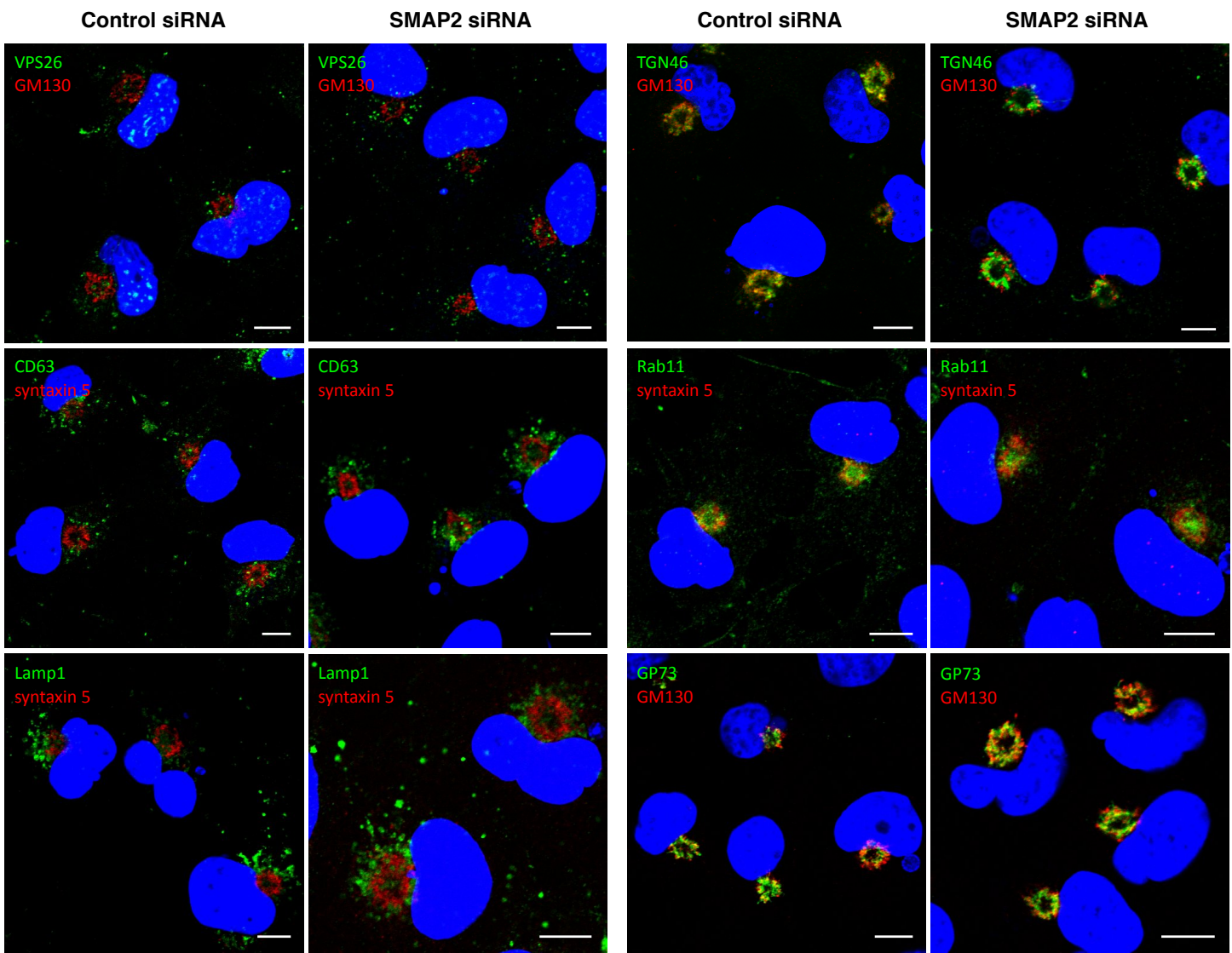

Supplement: Figure S3 — Cells treated with control siRNA or SMAP2 siRNA#1 for 72 h were fixed with PFA, permeabilized, and stained for GM130 (red), syntaxin 5 (red) or the indicated organelle markers (green). Scale bars, 10 µm. (PDF) [file pone.0069145.s003.pdf]
